# Opposing roles of two R-loop associated G-quadruplexes in tuning transcription activity

**DOI:** 10.1093/nar/gkaf930

**Published:** 2025-09-23

**Authors:** Leya Yang, Chun-Ying Lee, Tapas Paul, Sua Myong

**Affiliations:** Program in Cellular and Molecular Medicine, Boston Children’s Hospital, Harvard Medical School, Boston, MA 02115, United States; Program in Cellular and Molecular Medicine, Boston Children’s Hospital, Harvard Medical School, Boston, MA 02115, United States; Program in Cellular and Molecular Medicine, Boston Children’s Hospital, Harvard Medical School, Boston, MA 02115, United States; Program in Cellular and Molecular Medicine, Boston Children’s Hospital, Harvard Medical School, Boston, MA 02115, United States

## Abstract

Guanine (G)-rich sequences in nucleic acids can form non-canonical secondary structures such as R-loops and G-quadruplexes (G4) during transcription. The R-loop formed on the template strand promotes and stabilizes G4 in the non-template strand. However, the precise role of G4/R-loop-forming sequences on transcription remains poorly understood. In this study, we investigated the effect of different potential G4-forming sequences (PQSs) on G4/R-loop formation and transcription dynamics. We employed gel-based assays and single-molecule fluorescence resonance energy transfer (smFRET) to measure RNA synthesis and concomitant formation of G4 and R-loop during transcription by T7 RNA polymerase. We reveal two types of R-loop that form successively; an R-loop with an intramolecular DNA G4 (IG4) initially forms during transcription, followed by an R-loop with an intermolecular DNA:RNA hybrid G4 (HG4). We found that IG4 R-loops inhibit, whereas HG4 R-loops enhance transcription. We identified that an HG4/IG4 ratio highly correlates with transcriptional activity. PQS with short linkers favors IG4, reducing transcription, while PQS with long linkers that induce loosely folded PQS favor HG4, increasing transcription. Since IG4 formation precedes HG4, tightly folded PQS forms IG4 quickly and stably, slowing its conversion to HG4 and reducing transcriptional enhancement.

## Graphical Abstract

**Graphical Abstract:**
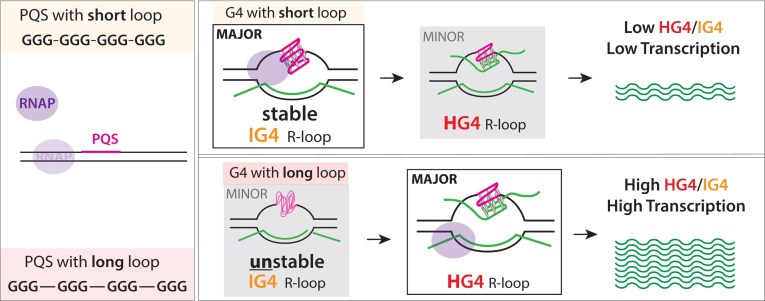

## Introduction

G-quadruplexes (G4s) and R-loops are non-canonical nucleic acid structures involved in cellular processes such as DNA replication, transcription, and translation [1–4] with implications for diseases including cancer [5–7] and neurological disorders [8, 9]. G4s, formed in guanine-rich sequences through Hoogsteen base pairing, result in four-stranded guanine-tetrad structures [10, 11]. Sequencing methods, such as G4-seq and G4-miner, have identified between 700 000 and 1 000 000 potential G4-forming sequences in the human genome [12, 13]. Computational studies and G4 ChIP-seq data reveal that G4 peaks are primarily enriched in regions upstream of the transcription start site (TSS), followed by enrichment in the 5′ UTRs [14–18]. Its presence correlates with gene expression through mechanisms such as modulating transcription factor binding and RNA polymerase activity [19–21].

R-loops are three-stranded nucleic acid structures that form in GC-rich regions during transcription, where a nascent RNA strand hybridizes with the DNA template strand, displacing the non-template strand. R-loops are not simply byproducts of transcription but play an active role in regulating gene expression by modulating transcription initiation, elongation, and termination [22–24]. DRIP-seq and R-ChIP studies have shown that R-loops are enriched in gene promoters of transcriptionally active genes [25–27]. However, persistent or misregulated R-loops are associated with DNA damage and genomic instability [2].

Recent studies have highlighted the functional interplay between G4s and R-loops [28]. The displaced, G-rich non-template strand within an R-loop can fold into a G4 structure, particularly in regions of high transcription activity and negative supercoiling, such as gene promoters and 5′ UTR. This interplay suggests the role of G4 and R-loop together in transcriptional regulation [29, 30].

In our previous study using a T7 RNA polymerase (T7 RNAP)-based *in vitro* transcription system [21], we investigated the potential G4-forming sequence (PQS) within the *c-MYC* proto-oncogene, which participates in key processes such as cell growth, differentiation, and apoptosis [31]. While PQSs are predominantly enriched in gene promoters [14], we focused on those located in the 5′UTR, which exhibits the second-highest level of PQS enrichment and remains relatively understudied [14]. Our goal was to explore the interplay between G4 and R-loop structures, rather than G4s alone. Since R-loops typically form downstream of promoter regions, the 5′UTR serves as a relevant context for studying these dynamics within our transcription system. We demonstrated that the R-loop forms during transcription when the *c-MYC* PQS is placed on the non-template strand within the 5′UTR. The R-loop exposes the PQS, enabling it to fold into a G4 structure, which is then stabilized. The simultaneous presence of G4 on the non-template strand and R-loop on the template strand promotes transcription through a mechanism involving successive rounds of R-loop formation [32].

Large-scale G4 and R-loop sequencing studies have correlated their presence upstream of the TSS and 5′UTR regions to enhanced gene expression [14, 15, 26, 27], and our *in vitro* studies have provided mechanistic insights into how PQS contributes to this in the 5′UTR region [33]. However, the effects of PQS sequence variations on these structures and their transcriptional outcomes remain poorly understood. G4s are defined by a core motif of four guanine tracts separated by loops of varying lengths and compositions. Variations in these motifs lead to structural diversity, influencing G4 stability and folding topology. For example, longer loops tend to increase structural flexibility [34, 35], while shorter loops impose constraints that enhance stability [36–38]. Although genomic surveys have ranked PQS sequences by loop length prevalence [39], the molecular mechanisms by which these variations regulate transcription in the 5′UTR are still not fully understood.

In this study, we investigate how PQS sequence variations modulate G4 and R-loop structures, which impact transcriptional outcomes. Using a T7 RNAP *in vitro* transcription system, electrophoretic mobility shift assays (EMSA), and single-molecule fluorescence resonance energy transfer (smFRET), we examined G4 and R-loop formation alongside RNA production across various PQS sequences placed downstream of the TSS to mimic the 5′UTR [40]. Our findings demonstrate that sequence variations impact transcription by generating two distinct G4/R-loop structures: an intramolecular G4 with R-loop (IG4 R-loop), which lowers transcription, and an intermolecular hybrid DNA:RNA G4 with R-loop (HG4 R-loop), which enhances transcription. The PQS sequence plays a role in determining the proportion of these structures: (i) shortest-loop PQS generates a high level of IG4 R-loop, which severely diminishes transcription; (ii) mid-length loop forms unstable IG4 R-loop, which gradually gives rise to HG4 R-loop, resulting in moderate level of transcription; and (iii) long-loop PQS favors the formation of HG4 R-loops, leading to higher transcriptional output. Therefore, the balance between HG4 R-loops and IG4 R-loops dictates transcriptional activity, highlighting the role of G4/R-loop structures in tuning transcription.

## Materials and methods

### DNA construct preparation

HPLC-purified DNA oligonucleotides containing biotin for immobilization and either Cy3, Cy5, or amine modifications were procured from Integrated DNA Technologies (IDT, USA). Amine-modified oligonucleotides were labeled with NHS ester-conjugated fluorescent dyes following established protocol [41, 42]. Duplex DNA constructs were prepared by mixing top, bottom, and biotin-conjugated 18-mer oligonucleotides at a molar ratio of 1:1.2:1.5. DNA strands were annealed in T50 buffer (10 mM Tris–HCl, pH 7.5, and 50 mM NaCl) using a thermocycler. The mixture was heated to 95°C for 2 min, then gradually cooled at a rate of 2°C/min until 40°C, followed by cooling at 5°C/min until 4°C [43]. The annealed constructs were stored at −20°C and freshly re-annealed before use. All buffers were prepared using Milli-Q water and filtered through 0.22 μm membrane filters.

### Single molecule FRET data acquisition and analysis

A home-built prism-type total-internal-reflection inverted fluorescence (TIRF) microscope (Olympus IX 71) was used for smFRET studies, as described previously [43–45]. DNA molecules were immobilized on polyethylene glycol (PEG)-passivated quartz slides via biotin–NeutrAvidin interactions. Transcription reactions were performed with 1 mM RNAP and 1 mM NTP mix in a buffer containing 40 mM Tris–HCl, pH 7.8, 50 mM KCl, 6 mM MgCl_2_, 1 mM DTT, 2 mM spermidine, and 0.1% bovine serum albumin. All smFRET measurements were carried out at room temperature (∼23°C ± 2°C) in an imaging buffer containing an oxygen scavenging system (10 mM trolox, 0.5% glucose, 1 mg/mL glucose oxidase, and 4 μg/mL catalase) to minimize photobleaching of the dyes [46, 47].

The evanescent field was generated through TIRF microscope using a solid-state 532 nm diode laser (Compass 315 M, Coherent) to excite the fluorophores in the sample chamber. Fluorescence from Cy3 (donor) and Cy5 (acceptor) was simultaneously collected using a water immersion objective and projected onto an EMCCD camera (Andor) after passing through a dichroic mirror (cutoff = 630 nm). Data were recorded with a time resolution of 100 ms and analyzed using scripts written in Interactive Data Language (IDL) (http://www.exelisvis.co.uk/ProductsServices/IDL.aspx) and MATLAB (https://www.mathworks.com/).

The FRET efficiency (*E_FRET_*) was calculated using *I_A_*/(*I_D_* + *I_A_*), where *I_D_* and *I_A_* represent the intensity of donor and acceptor, respectively. FRET histograms were generated from more than 4000 molecules (21 frames from 20 short movies) across different imaging surfaces. Alternating green and red laser excitation (10 frames each, separated by a dark frame) excluded donor-only molecules in low EFRET regions. Donor leakage was corrected based on donor-only E_FRET_ values. Histograms were normalized and fitted with multi-peak Gaussian distributions. Long movies (2000 frames, i.e. 200 s) were recorded to observe molecular behavior [42, 46].

### *In vitro* T7 transcription assay

Transcription reactions were carried out in a total volume of 20 μL containing the following components: RNAP transcription buffer (40 mM Tris–HCl pH 8.3, 50 mM KCl, 6 mM Mg_2_Cl, 2 mM spermidine, 1 mM dithiothreitol), 50 mM KCl, RNase Inhibitor Murine (200 U/mL, NEB), T7 RNAP (1250 U/mL, NEB), and 10 nM DNA template. For transcription in different salt conditions, the 50 mM KCl was replaced with 50 mM of LiCl or water for the no monocation condition.

Transcription was initiated by adding 1 mM rNTPs. For inosine triphosphate (ITP) transcription, where GTP was substituted with ITP, the reaction was initiated with 1 mM ATP, CTP, UTP, 1 mM ITP, and 4 mM GMP (guanosine monophosphate). Similarly, for transcription with 7-deaza-rGTP, where GTP was replaced by 7-deaza-rGTP, the reaction was initiated with 1 mM ATP, CTP, UTP, 1 mM 7-deaza-rGTP, and 4 mM GMP.

The reaction mixture was incubated at 25°C for the desired transcription duration. Transcription was terminated by first adding 0.5 μL of 0.5 M ethylenediaminetetraacetic acid (EDTA), then 4 μL of solution containing 50% glycerol and 10% sodium dodecyl sulfate (SDS) in the ratio 20:1, respectively.

### Transcription with pre-folded G-quadruplex

To pre-fold G4, the DNA was annealed in a solution containing 10 mM Tris–HCl, 100 mM KCl, and 40% PEG-200. The DNA was annealed by heating to 95°C and gradually cooling at a rate of 1°C per minute until reaching 25°C. Once the pre-folding process was complete, transcription was carried out as described in the previous section.

### RNase H digestion

To terminate transcription prior to RNase H digestion, 2 μL of 10 μM T7 promoter DNA was added to competitively inhibit T7 RNAP. Following transcription termination, 2.5 units of RNase H (NEB) were added to the reaction mixture. The sample was incubated at 25°C for 5 min to allow for digestion. Digestion was terminated by adding 0.5 μL of 0.5 M EDTA, 4 μL of 50% glycerol, and 0.1 μL of 10% SDS.

### RNase A digestion

RNase A (10 mg/mL) was diluted 1:1000 in T50 buffer (10 mM Tris–HCl, 50 mM NaCl). Prior to transcription termination, 1 μL of the diluted RNase A was added to the transcription reaction and incubated at 25°C for 10 min. The reaction was then terminated by adding 0.5 μL of 0.5 M EDTA followed by 4 μL of a 20:1 mixture of 50% glycerol and 10% SDS.

### Electrophoretic mobility shift assay

All EMSAs were performed using a 10% polyacrylamide gel. The gel composition included 10% acrylamide/bis-acrylamide solution (29:1), 1× TBE buffer, 1% ammonium persulfate, and 1% TEMED. A total of 7 μL of transcribed samples and the low molecular weight ladder (New England Biolabs) were loaded into each gel, and electrophoresis was performed at 4°C under a constant current of 12 mA per gel for 1 h. After electrophoresis, the gel was stained for 10 min in 0.5× SYBR™ Green II RNA gel stain, prepared by diluting 2.5 μL of 10 000× SYBR™ Green II RNA Gel Stain concentrate in 50 mL deionized water, and subsequently destained in deionized water for 5 min. Imaging was conducted using the Azure 400 Imager, with Cy5 fluorescence detected at 628 nm for 3 min to visualize the DNA template and SYBR™ Green II fluorescence detected at 472 nm for 3 s to visualize RNA.

### FRET gel imaging

Prior to SYBR Green II RNA staining, the gel was imaged at Cy3 excitation fluorescence and Cy5 emission fluorescence for 1 min.

### Gel quantification

Quantification of gels was performed using ImageJ software. The intensities of the IG4 and HG4 bands were determined from the Cy5 signals corresponding to their shifted positions on the gel. These intensities were normalized to the total Cy5 signal in the respective lane to calculate the fraction of IG4 and HG4 relative to the total DNA signal. For RNA quantification, RNA bands were identified as those with faster mobility than the linear DNA template band and exhibiting SYBR™ Green II fluorescence without Cy5 signal. This distinction was made because SYBR™ Green II dye also stains DNA. The intensities of these RNA bands were summed and normalized to the combined SYBR™ Green II signal of DNA, IG4, and HG4 in the same lane. Uncropped gel images and corresponding normalized quantification values are provided in the data source file.

### Data analysis

For transcription rate measurements, RNA band intensities were quantified from the SYBR™ Green II fluorescence signals. They were normalized to the total nucleic acid signal in each lane by dividing the RNA intensity by the combined intensities of DNA, IG4, and HG4 bands. RNA levels were then plotted over time (0, 0.5, 3, 5, 10, 15, 20, and 30 min post transcription). A linear regression was applied to determine the transcription rate, with the slope representing the rate. For kinetic modeling, the normalized intensities of the DNA, IG4 R-loop, and HG4 R-loop bands were used as inputs to the chem_kin Python fitting package. All source code is provided in the “Data Availability” section.

### Statistical analysis

For all quantified gels, at least three independent replicates were performed. The mean values were plotted, and error bars represent the standard deviation of the three replicates. Two-tailed t-tests were used to calculate *P*-values, with statistical significance defined as *P* < .05. Raw data and exact *P*-values are provided in the accompanying data source file.

## Results

### Two distinct R-loops form in PQS during transcription

**Figure 1.**
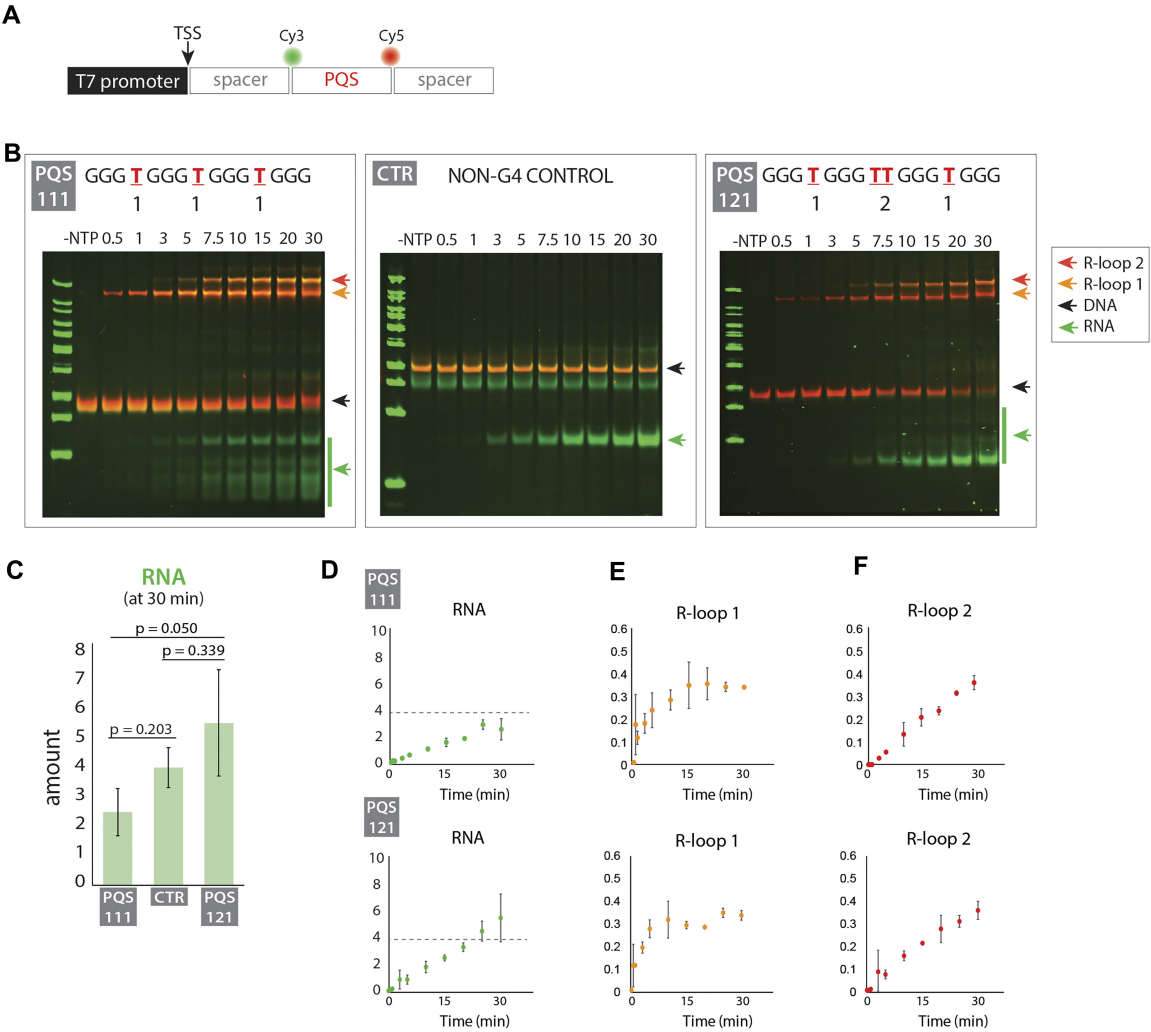
Formation of two distinct R-loops during transcription of PQS-containing DNA sequences. (**A**) Schematic of the DNA construct containing a T7 promoter, a 16 base pair spacer, a PQS region flanked by Cy3 and Cy5 dyes, and a 14 base pair spacer downstream of the PQS. (**B**) EMSA gel showing transcription of PQS 111, a non-G4 control sequence, and PQS 121, from initiation to 30 min after the addition of transcription components. This gel is a merged overlay with red indicating DNA bands and green indicating RNA bands. PQS sequences are named based on linker length. (**C**) Quantification of RNA levels from the EMSA at the 30-min time point for PQS 111, the non-G4 control sequence, and PQS 121. (**D**–**F**) Quantification of EMSA results over transcription time for PQS 111 and PQS 121, showing the relative amounts of DNA, R-loop 1, and R-loop 2 bands. Data represent the mean ± standard deviation from three independent replicates. Raw values are provided in the data source file. The gray horizontal line indicates RNA levels from the non-G4 control sequence after 30 min of transcription.

To investigate G4 and R-loop formation during transcription of varying PQS, we designed a DNA construct containing a T7 promoter and a PQS on the non-template strand positioned downstream of the TSS after a 16-bp spacer sequence. We focused on the non-template strand because our previous study demonstrated that PQS on the non-template strand positively influences transcription [21]. To monitor R-loop and G4 formation, we labeled the 5′ and 3′ ends of the PQS sequence with a Cy3 and Cy5, respectively, as done previously (Fig. 1A). The constructs were named based on the number of thymidine linkers between the runs of guanine. For example, 111 is characterized by one thymidine linker between each G-run, i.e. [GGG T GGG T GGG T GGG], while 123 refers to [GGG T GGG TT GGG TTT GGG].

We performed *in vitro* transcription using T7 polymerase and monitored time-dependent G4/R-loop formation and RNA production through EMSA. Due to the increased hydrodynamic radius and molecular weight, R-loops were observed as upshifted bands with reduced mobility compared to linear DNA [21]. Cy5 fluorescence allowed us to directly visualize DNA constructs, while RNA was detected using SYBR Green II RNA staining.

Before transcription initiation, the linear DNA band for PQS 111 was observed as a single band exhibiting a Cy5 fluorescence signal. After adding rNTPs to initiate transcription, two distinct low-mobility bands appeared. We designated these as R-loop 1, which forms first and exhibits slightly higher mobility than R-loop 2, which forms later and shows lower mobility (Fig. 1B). In our previous study using the PQS-cMyc sequence [GGG T GGG TA GGG T GGG], only a single upshifted G4/R-loop band was observed. However, in this study, PQS 111 and PQS 121 produced two distinct R-loop bands, particularly noticeable when the gel-running duration was extended (Fig. 1B, left). Concurrently, RNA production was detected, with bands increasing in intensity over time. Transcription using a control DNA construct, which lacked a PQS sequence but contained a scrambled sequence with 50% GC density, produced RNA but no R-loop bands (Fig. 1B, middle), confirming that PQS is necessary for R-loop formation.

We compared transcription outcomes between PQS 111 and PQS 121, which has a similar linker length to the PQS found in the *c-MYC* promoter sequence from our previous study, but with a TT linker in the middle instead of TA. Both PQS 111 and 121 produced G4/R-loops and formed two distinct R-loop structures during transcription (Fig. 1B, left and right), suggesting that the presence of two R-loops is a common feature of PQS-mediated transcription. We observed that transcripts from the PQS-containing sequences appeared as smeared RNA bands, while the control sequence produced a single, distinct RNA band. This smearing likely reflects secondary structures formed by G-rich RNA. A denaturing gel revealed two distinct RNA bands (Supplementary Fig. S1A): one corresponding to the expected full-length RNA and another with lower mobility, likely due to G-rich RNA folding.

Quantitative analysis showed that after 30 min of transcription, the 111 PQS construct produced less RNA compared to both the control sequence and the 121 PQS construct, indicating that even one nucleotide difference in the loop length can have an impact on transcription yield (Fig. 1C). RNA production kinetics differed between the two constructs, with PQS 111 exhibiting a lower RNA production rate compared to PQS 121 (Fig. 1D). Interestingly, the R-loop 1 and 2 formation dynamics differed between the two constructs. R-loop 1 was similar in both constructs, with PQS 111 plateauing at around 15 min while PQS 121 peaking at around 10 min before decreasing after 20 min (Fig. 1E). R-loop 2 showed a continuous linear increase in both constructs, with PQS 121 displaying a higher formation rate, reaching a higher level earlier than PQS 111 (Fig. 1F).

These findings demonstrate that PQS sequences influence the kinetics of two different R-loop formations and RNA production. We hypothesize that R-loop 1 and R-loop 2 levels that arise from the variations in PQS sequences are responsible for different transcription outcomes.

### Formation of two R-loops either with intramolecular or hybrid G-quadruplexes

We conducted a series of tests to identify R-loop 1 and R-loop 2. Our previous study demonstrated that R-loops form before G4 and that G4 formation stabilizes R-loop structure. Together, G4 and R-loops coexist to facilitate transcription.

**Figure 2.**
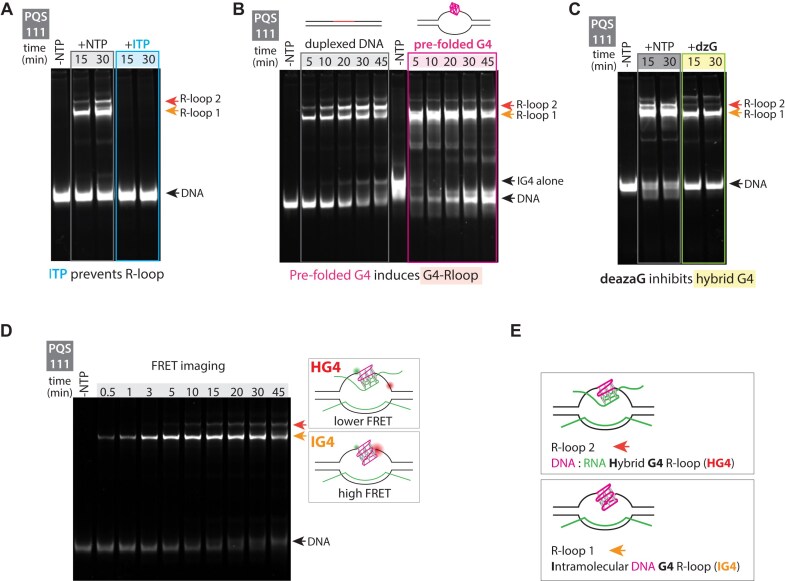
Identification of R-loop 1 and R-loop 2 as distinct G4-associated structures. R-loop 1 is composed of an intramolecular DNA G4 and an R-loop, while R-loop 2 consists of a DNA:RNA hybrid G4 structure with an R-loop. (**A**) EMSA comparing transcription under normal conditions with rNTPs versus with ITP substituted for rGTP to assess R-loop formation. (**B**) EMSA comparing transcription using normal linear duplex DNA versus pre-folded G4 DNA. (**C**) EMSA comparing transcription under normal conditions with rNTPs versus with 7-deaza-rGTP substituted for rGTP to test for DNA:RNA hybrid G4 formation. (**D**) Gel imaged using FRET, showing Cy3 excitation and Cy5 emission to distinguish the IG4 band. (**E**) Schematic representation of R-loop 1 as an intramolecular DNA G4 R-loop structure and R-loop 2 as a DNA:RNA hybrid G4 R-loop structure.

To verify the identity of R-loop 1 and R-loop 2, we treated the samples with RNase H, which specifically digests DNA:RNA hybrids. Following treatment, R-loop 2 completely disappears, while R-loop 1 is partially reduced, suggesting it may be more resistant to RNase H cleavage (Supplementary Fig. S1B). To further verify whether R-loop 1 and R-loop 2 contain R-loops, we substituted GTP with ITP during transcription. ITP inhibits R-loop formation due to the reduced stability of inosine-cytosine base pairing [48–50]. Transcription with ITP eliminated both R-loop 1 and R-loop 2, indicating that both bands are R-loops (Fig. 2A).

Next, we tested whether R-loop 1 or R-loop 2 contains an IG4, where the PQS in the non-template strand folds into a G4 while the DNA:RNA hybrid of the R-loop forms on the template strand, i.e. IG4/R-loop. We define the IG4 R-loop as a G4 R-loop structure formed exclusively by G-runs within the DNA. Based on our previous finding that pre-folded G4 in non-template DNA leads to fast and high levels of IG4/R-loop, we compared transcription activity between a normal duplex DNA versus the construct with a pre-folded G4 structure. Before transcription initiation, i.e. NTP, the pre-folded G4, due to its bulkier secondary structure, exhibited slightly lower mobility than the linear DNA construct (Fig. 2B, right side). As the transcription progressed, pre-folded G4 generated a prominent R-loop 1 band with no formation of R-loop 2 (Fig. 2B, right). The predominant formation of the R-loop 1 band under pre-folded G4 conditions indicates that R-loop 1 contains IG4. Additionally, the R-loop 1 band exhibited a significantly higher shift than the IG4-alone band (Fig. 2B, right gel, -NTP), further supporting the presence of IG4 associated with the R-loop (Fig. 2B).

Based on previous studies and the lower mobility shift of R-loop 2, we conjectured that R-loop 2 could contain an intermolecular hybrid G4 (HG4), formed by the G-rich non-template DNA strand pairing with the transcribed G-rich RNA [51–53]. This hybrid structure likely arises because the transcribed RNA shares the same G-runs as the non-template DNA strand. These G-runs in the RNA can potentially form Hoogsteen base pairs with the G-runs in the PQS sequence of the non-template DNA strand, forming an intermolecular hybrid DNA:RNA G4 (HG4). We define the HG4 R-loop as a G4 R-loop structure formed by G-runs contributed by both DNA and RNA strands. To test for HG4, we replaced rGTP with deaza-rGTP, an rGTP analog that cannot form the hydrogen bonds required for G4 formation. Therefore, transcription with deaza-rGTP produces RNA with G-runs incapable of participating in G4 formation with the DNA strand, thereby preventing HG4 formation [53]. Transcription with deaza-rGTP eliminated R-loop 2, while R-loop 1 remained, indicating that R-loop 2 contains an HG4 structure along with the R-loop (Fig. 2C).

Furthermore, we performed FRET imaging of the gel to distinguish between the IG4 R-loop and HG4 R-loop. Our DNA construct includes Cy3 donor and Cy5 acceptor dyes flanking the PQS sequence. When IG4 forms, the reduced distance between the two dyes is expected to produce higher FRET than HG4. Excitation of Cy3 fluorescence and detection of Cy5 signals revealed a higher FRET intensity in the R-loop 1 band compared to R-loop 2, further confirming R-loop 1 and R-loop 2 as IG4 R-loop and HG4-containing R-loop, respectively (Fig. 2D).

To rule out the possibility that a head-to-head dimeric IG4 may form [54], which comprises one DNA and one RNA strand, we performed transcription using the 1 construct i.e. GGGTGGG, which cannot form any type of G4. When transcription was carried out with the 1 construct, we observed no low-molecular-weight bands (Supplementary Fig. S2A), indicating that head-to-head IG4 dimers are not forming under these conditions.

We repeated these structural experiments to determine the identity of R-loop 1 and R-loop 2 bands with additional PQS constructs, and the results were consistent across all constructs. R-loop 1 was prominent when pre-folded DNA constructs were used, while R-loop 2 disappeared (Supplementary Fig. S2B), supporting the identity of R-loop 1 as an IG4 R-loop. In experiments where rGTP was replaced with 7-deaza-rGTP, R-loop 2 diminished across constructs, consistent with it being an HG4 R-loop (Supplementary Fig. S2C). FRET gel imaging also showed strong signal at the R-loop 1 position, further supporting its assignment as an IG4 R-loop (Supplementary Fig. S2D). These findings demonstrate that R-loop 1 is an IG4 R-loop that forms first during transcription, followed by R-loop 2, which is the DNA:RNA HG4 R-loop (Fig. 2E). However, the specific role of these structures in regulating transcription remains unclear.

### IG4 R-loop inhibits while HG4 R-loop enhances transcription

Having assigned the two types of R-loops, we sought to investigate the roles of IG4 R-loop and HG4 R-loop in transcription by choosing experimental conditions that favor IG4 R-loop or HG4 R-loop formation and measuring the corresponding RNA output. We used the 121 PQS construct for comparison, as it yields higher RNA levels than 111, providing a more reliable baseline for detecting transcriptional changes.

**Figure 3.**
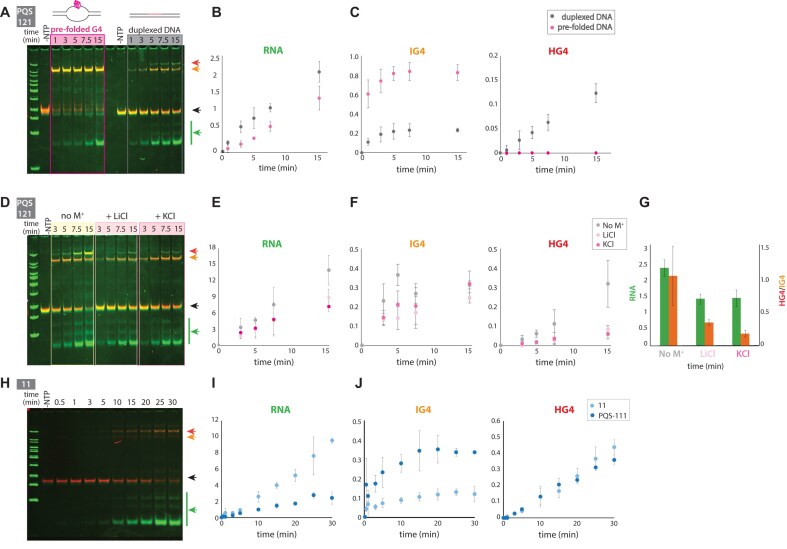
IG4 R-loop reduces while HG4 R-loop facilitates transcription. (**A**) EMSA comparing transcription using normal linear duplex DNA versus pre-folded G4 DNA. (**B**, **C**) Quantification of RNA, IG4, and HG4 levels from the EMSA in panel (A), comparing transcription using normal linear duplex DNA versus pre-folded G4 DNA. (**D**) EMSA comparing transcription under different ionic conditions: transcription buffer without monovalent cations, 50 mM LiCl, or 50 mM KCl. (**E**, **F**) Quantification of RNA, IG4, and HG4 levels from the EMSA in panel (D), comparing transcription under varying salt conditions. (**G**) Two-axis plot showing the correlation between RNA levels and the HG4/IG4 ratio under different ionic conditions. (**H**) EMSA of the PQS 11 construct, which contains 3 runs of G separated by a single thymidine linker, compared to PQS 111. (**I**, **J**) Quantification of RNA, IG4, and HG4 levels from the EMSA, comparing transcription between PQS 111 and PQS 11 constructs. All data represent the mean ± standard deviation from three independent replicates. Raw values are available in the source data file. EMSA gels in panels (A), (D), and (H) are merged overlays with DNA bands shown in red and RNA bands in green.

As before, we used pre-folded G4 to favor IG4 R-loop formation during transcription [21, 55, 56]. Under this condition, RNA production was reduced compared to the linear PQS construct (Fig. 3A and B). As expected, pre-folded G4 promoted significantly higher IG4 formation than the linear PQS construct and eliminated HG4 formation compared to the linear PQS condition (Fig. 3C). These findings suggest that IG4 formation lowers transcription but does not completely block it.

To further test the role of IG4 R-loop, we modulated IG4 stability by performing transcription in solutions containing no monovalent cation, LiCl, or KCl, listed in order from the least to the most IG4-stabilizing conditions [57–60]. In the absence of monovalent cations, IG4 stability was reduced, as indicated by a decrease in IG4 band intensity over longer transcription time (Fig. 3D). Concurrently, RNA production was increased compared to LiCl- and KCl-containing conditions, confirming that IG4 reduces transcription yield (Fig. 3E). The slight difference in RNA production between LiCl and KCl suggests that the 121 PQS sequence forms relatively stable IG4 even in LiCl conditions. While the IG4 levels remained similar across all three conditions, HG4 levels varied significantly, with the no-monovalent cation condition yielding the highest HG4 formation (Fig. 3F). This indicates that destabilizing IG4 may promote HG4 formation and that HG4 may enhance transcription.

Since the IG4 and HG4 R-loops play opposite roles in transcription, we reasoned that the HG4/IG4 ratio can be a helpful index that may be correlated to transcription output. Indeed, we observed a positive correlation between the HG4/IG4 ratio and RNA production (Supplementary Fig. S3A). Likewise, the increased presence of HG4 in the no-monovalent cation condition raised the HG4/IG4 ratio, which correlated with higher RNA production. These results suggest the role of HG4 in facilitating transcription (Fig. 3G).

To isolate the effect of HG4 R-loop, we used the “11” construct (-GGGTGGGTGGG-), which contains only three G-runs. Since four G-runs are required for IG4 formation, the 11 construct cannot form IG4, but can still form HG4 through Hoogsteen base pairing between G-runs in the RNA and the G-rich non-template DNA strand. Strikingly, the 11 construct produced significantly more RNA than the 111 PQS construct (Fig. 3H and I) with an increased HG4/IG4 ratio (Supplementary Fig. S3B). The IG4 levels were higher in the 111 PQS construct, while HG4 levels were similar between the two constructs (Fig. 3J).

These findings suggest that transcriptional output is influenced by the ratio between HG4 and IG4, with higher IG4 R-loop levels associated with reduced RNA production and higher HG4 R-loop levels promoting increased RNA production.

### RNA production positively correlates with PQS loop length and HG4/IG4 ratio

**Figure 4.**
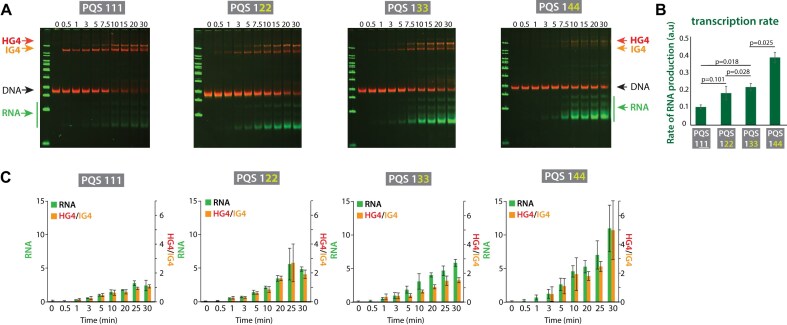
Longer linker PQS increases RNA production. (**A**) EMSA gels showing transcription of PQS 111, 122, 133, and 144, each containing progressively longer linker lengths. EMSA gels are merged overlays with DNA bands shown in red and RNA bands in green. (**B**) Quantification of transcription rate. The transcription rate was determined from the slope of RNA intensity over time, based on gel quantification. Two-tailed t-test was used to calculate statistical significance. (**C**) Two-axis plots showing the correlation between RNA levels and the HG4/IG4 ratio for each construct, demonstrating a similar pattern of increase with longer linker lengths.

To investigate whether the loop length of PQS sequences affects transcription, we measured transcription using 111, 122, 133, and 144 PQS constructs, each with progressively longer thymine linker lengths (Fig. 4A). We observed that increasing the linker length corresponded to increased RNA production (Supplementary Fig. S4A) and transcription rate (Fig. 4B). A positive correlation between the HG4/IG4 ratio and RNA production was consistent across the constructs. PQS 111, with the shortest linker, produced the least RNA and exhibited the lowest HG4/IG4 ratio, while PQS 144, with the most extended linker, showed the highest RNA production with the highest HG4/IG4 ratio (Fig. 4C). These results suggest that longer linker lengths may destabilize IG4 R-loop formation, shifting the balance toward HG4 R-loop. The increased HG4/IG4 ratio amplifies HG4’s promoting effect on transcription, driving higher RNA production.

### Single molecule analysis reveals unstable IG4 R-loops in longer loop lengths

**Figure 5.**
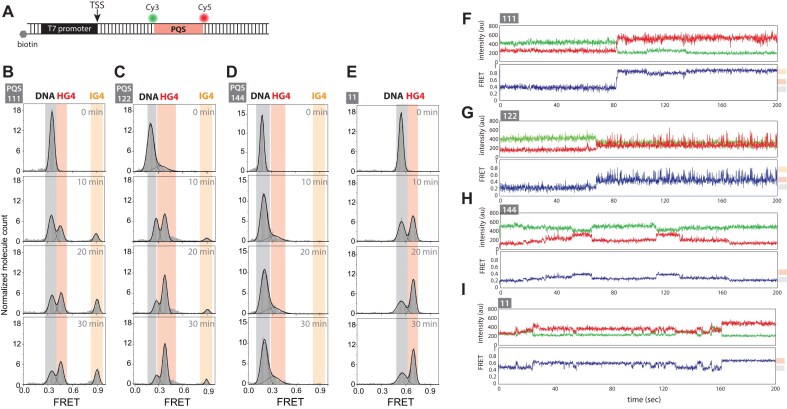
Single molecule data reveals unstable IG4 formation in PQS with longer loop length. (**A**) Schematic of the single molecule DNA construct, featuring a biotinylated strand for surface tethering, T7 promoter, a 16-bp spacer, a PQS region flanked by Cy3 and Cy5 dyes, and a 14-bp spacer downstream of the PQS. (**B**–**E**) smFRET histograms indicating high-, mid-, and low-FRET peaks, corresponding to IG4, HG4, and DNA. (**F**–**I**) Representative traces for PQS 111, 122, 133, and 144.

Next, we sought to probe the loop length dependence in terms of IG4- and HG4 R-loop formation by smFRET analysis. We used the same set of DNA constructs with one biotinylated strand for surface tethering (Fig. 5A). As before, the Cy3 and Cy5 dyes are positioned at either end of the PQS sequence downstream of the TSS. The FRET histogram is plotted by collecting FRET values from over 4000 molecules. Before transcription, the FRET peak is ∼0.3 for 111 and 122 (Fig. 5B and C, DNA). For 144 and 11, the FRET peak is ∼0.2 and ∼0.6, respectively, corresponding to longer and shorter linker lengths (Fig. 5D and E, DNA).

The FRET histograms taken after 10, 20, and 30 min of transcription show a progressive shift from low to higher FRET states to varying degrees depending on the linker length (Fig. 5B–E, mid-, high FRET). We interpret the mid-FRET as representing the R-loop without any G4 and HG4 R-loop, while the high FRET signal corresponds to the IG4 R-loop, based on ensemble gel-based FRET analysis (Fig. 2E) and our previous study [21]. As demonstrated previously, the R-loop without G4 is mostly a short-lived transient state; thus, the HG4 R-loop likely contributes more to the mid-FRET peak.

The high FRET IG4 R-loop forms the most in 111, followed by 122, with no formation in 144 and 11. In contrast, the HG4 R-loop forms far less in 111 than in 122 and 11. The mid-FRET state is not well distinguished in 144 due to the long dye-to-dye distance. The IG4- and HG4 R-loop pattern is consistent with the ensemble results, which displayed the lowest HG4/IG4 ratio of 111, which produces the least transcript.

Representative smFRET trace for 111 shows the low FRET shifting first to a transient mid-FRET, which leads to a high FRET, representing a transient R-loop followed by an immediate IG4 folding (Fig. 5F). Strikingly, 122 displays a transition from a low to a mid-FRET R-loop followed by a rapid FRET fluctuation that continues for a long duration, frequently exchanging between the R-loop and IG4 R-loop state, likely due to a less stable IG4 in 122 (Fig. 5G). This contrasts with the steady high FRET state, i.e. IG4 R-loop seen in 111, which forms a highly stable IG4. For 144 and 11, the FRET level switches between a long-lived low FRET and mid-FRET without transitioning to high FRET, consistent with the prominent HG4 and the lack of IG4 R-loop (Fig. 5H and I). Together, we demonstrate that the IG4 R-loop stability is a primary factor influencing transcription output. Highly stable IG4 R-loop lowers HG4 formation, thereby reducing transcription, whereas unstable IG4 can give rise to HG4, enhancing transcription.

### RNA production is independent of linker positions

**Figure 6.**
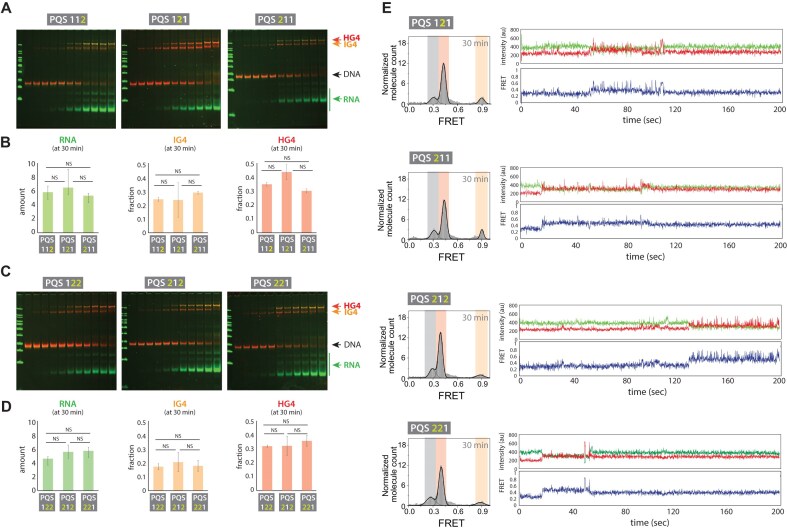
Linker position does not contribute to transcription. (**A**) EMSA analysis of transcription for PQS 112, 121, and 211, where the increased linker length is positioned at different regions. EMSA gels are merged overlays with DNA bands shown in red and RNA bands in green. (**B**) Quantification of RNA, IG4, and HG4 levels for PQS 112, 121, and 211. (**C**) EMSA analysis of transcription for PQS 122, 212, and 221, where the increased linker length is positioned at different regions. (**D**) Quantification of RNA, IG4, and HG4 levels for PQS 122, 212, and 221. (**E**) smFRET histogram and representative traces for PQS 121, 211, 212, and 221. Quantification in panels (B) and (D) is based on three independent replicates; data are presented as mean ± standard deviation. Two-tailed t-tests were used to assess statistical significance (*P* > .05). Raw data are provided in the accompanying source file.

To determine whether the position of loop length variation within the PQS sequence affects transcription, we performed *in vitro* transcription with PQS 112, 121, and 211 constructs. We observed no differences in RNA production, IG4 formation, or HG4 formation across these constructs (Fig. 6A and B). Similarly, transcription experiments with PQS 122, 212, and 221 also showed no changes in RNA production, IG4, or HG4 levels (Fig. 6C and D). These results indicate that varying the loop positions within the PQS sequence does not influence transcription.

We conducted single molecule measurements on four constructs, 121, 211, 212, and 221, all containing 1 and 2 loop lengths in different positional combinations (Fig. 6E). The results reveal that all constructs behave similarly. First, they all generate a lower level of IG4 R-loop and a higher level of HG4 R-loop, consistent with the EMSA analysis. The difference between the IG4 R-loop and HG4 R-loop is slightly less evident in EMSA, likely due to the constricting gel matrix that may stabilize the IG4 R-loop state. Second, they exhibit fluctuating FRET, indicative of an unstable IG4 R-loop that oscillates between a plain R-loop and an IG4 R-loop, like the case of 122 (Fig. 5G).

### Regulation of RNA production by competing IG4 and HG4 formation

**Figure 7.**
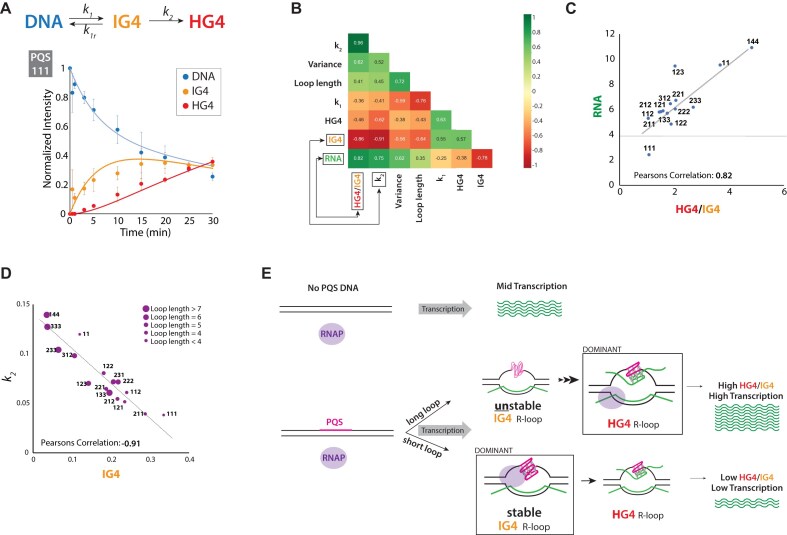
Competing dynamics between HG4 and IG4 determine transcription outcome. (**A**) Proposed model that best fits the data, where DNA reversibly transitions to IG4 with forward (*k*_*1*_) and reverse (*k_1r_*) rate constants. IG4 then irreversibly transitions to HG4 at rate *k*_*2*_. Fitting of the quantified levels of DNA, IG4, and HG4 for PQS 111 using the model. (**B**) Correlation plot illustrating relationships between different variables based on data from various PQS sequences. “Variance” represents the variation in loop length at different sequence positions. *k*_*1*_ and *k*_*2*_ are rate constants derived from fitting the model to experimental data. (**C**) Plot showing a strong positive correlation between the HG4/IG4 ratio and RNA production, with specific PQS sequences labeled at corresponding data points. The gray horizontal line indicates RNA levels produced by the non-G4 control sequence at 30 min. (**D**) Plot showing a strong negative correlation between IG4 levels and *k*_*2*_, with specific PQS sequences labeled. Marker size represents loop length, with shorter loops depicted as smaller markers and longer loops as larger markers. (**E**) Summary schematic illustrating how transcription of PQS sequences with varying loop lengths leads to either a dominant IG4 or HG4 structure, ultimately influencing transcriptional outcomes.

To investigate how the kinetics of IG4 and HG4 formation affect transcription across varying PQS sequences, we sought to develop a simple model to explain their formation dynamics during transcription. After testing several models, we found the best fit to be ${\mathrm{DNA}} \rightleftharpoons {\mathrm{IG}}4 \rightarrow {\mathrm{HG}}4$ (Supplementary Fig. S5A–C). This model accurately describes our data (Fig. 7A).

To further test the roles of IG4 R-loop and HG4 R-loop in RNA production, we conducted *in vitro* transcription with additional PQS DNA constructs featuring varying loop lengths (Supplementary Table S1). Ranking RNA production across these constructs revealed differences in RNA output and IG4 and HG4 amounts (Supplementary Fig. S6A–C). To determine the driving factor(s) responsible for the differences, we analyzed correlations between RNA production and several parameters: HG4 R-loop, IG4 R-loop, loop length variance, loop length, and the kinetic parameters *k_1_* (rate of IG4 formation) and *k_2_* (rate of HG4 formation). Among the pairwise correlations, the HG4/IG4 ratio shows the strongest positive correlation with RNA production, followed by *k_2_* (Fig. 7B). In contrast, IG4 displayed the strongest negative correlation with RNA production. HG4 alone did not strongly correlate with RNA output; the HG4/IG4 ratio better explains transcriptional enhancement, with higher HG4 relative to IG4 associated with increased RNA production (Fig. 7C).

We also observed a strong negative correlation between IG4 levels and *k_2_*, suggesting that stable IG4 has lower propensity to convert to HG4. Since HG4 facilitates transcription, strong IG4 formation lowers transcriptional enhancement by limiting its conversion to HG4. Examining sequence features more closely, we found that PQS constructs with longer loop lengths generally exhibited reduced IG4 levels and higher *k_2_* values (Fig. 7D). This further supports the idea that longer loop lengths weaken IG4 stability, facilitating its conversion to HG4 and promoting transcriptional enhancement.

To further assess IG4 R-loop stability, we calculated the equilibrium constant (*k*_1_/*k*_1r_) and ranked PQS sequences accordingly (Supplementary Fig. S7A). IG4 R-loops exhibited varying degrees of resistance to RNase H digestion depending on the PQS sequence, leading us to use RNase H resistance as a proxy for IG4 stability. Plotting IG4 R-loop band intensity after RNase H digestion against the equilibrium constant (*k*_1_/*k_1r_*) revealed a positive correlation, indicating that IG4 R-loops with shorter loop lengths are more resistant to RNase H digestion and have higher *k*_1_/*k*_1r_ values, whereas those with longer loop lengths show the opposite trend (Supplementary Fig. S7B).

Furthermore, as loop length increases, we observe a negative correlation between the ratio of IG4 remaining after RNase H digestion and total IG4 before RNase H digestion (Supplementary Fig. S7C-D). This supports the idea that IG4 stability decreases with increasing loop length. These findings further confirm that PQS loop length influences IG4 R-loop stability and suggest that weaker IG4 R-loop structures facilitate their conversion to HG4 R-loops, ultimately promoting transcriptional enhancement.

### IG4 R-loop reduces transcription by trapping RNAP

We asked how IG4 R-loop reduces transcription by running an EMSA with and without SDS treatment. While the SDS-treated sample removes the protein, allowing for probing the nucleic acid structure in the absence of protein, the non-SDS-treated sample (EDTA only) retains the protein-nucleic acid interaction. The non-SDS-treated samples exhibited bands that appear on top of the IG4- and HG4 R-loop, suggesting a higher-order complex (Supplementary Fig. S8A). When the same samples were treated with SDS, the upper bands disappeared and shifted downward to the IG4 R-loop bands (Supplementary Fig. S8B and C), strongly suggesting that the extra upper bands in the no-SDS condition arise from the IG4 R-loop complexed with RNAP. Notably, the HG4 R-loop band remained unchanged in both conditions, suggesting that HG4 R-loops are not involved in the same RNAP-associated complexes as IG4 R-loops (Supplementary Fig. S8C).

We conclude that the loop length of PQS modulates transcription outcome through two different R-loops: the IG4 and HG4 R-loops. When the PQS possesses a short loop length, such as 111, transcription activity is highly prone to form an IG4 R-loop due to the stability of the G4 structure in the non-template DNA. The IG4 R-loop structure traps RNAP, likely lowering the transcription activity by preventing successive RNAP loading. In contrast, long loop length in PQS leads to an unstable IG4 R-loop, which resorts to the HG4 R-loop as more RNA is produced. The HG4 R-loop facilitates transcription, likely by opening the transcription bubble and promoting RNAP translocation (Fig. 7E).

## Discussion

Our study investigated the effects of various PQS sequences on transcription, identifying two distinct R-loop-associated G4 structures. During transcription, an intramolecular G4 with an R-loop (IG4 R-loop) forms first, followed by an intermolecular hybrid DNA:RNA G4 with an R-loop (HG4 R-loop) (Figs 1 and 2). These structures have opposing roles in transcription: IG4 R-loops limit RNA production, while HG4 R-loops enhance it. To confirm HG4’s transcription-enhancing role, we destabilized IG4 R-loops in weak or no monovalent cation conditions and observed enhanced RNA production. Additionally, using the 11 PQS sequence, which exclusively forms HG4 R-loops, we recorded high RNA output, further validating HG4’s positive impact on transcription (Fig. 3). Overall, RNA production is modulated by the IG4 and HG4, with a higher HG4/IG4 ratio correlating strongly with increased RNA output (Fig. 4).

To understand the formation kinetics of IG4 R-loop and HG4 R-loops, we tested different models and found that the ${\mathrm{DNA}} \rightleftharpoons {\mathrm{IG}}4 \rightarrow {\mathrm{HG}}4$ model best fit our data. We also considered an alternative model (${\mathrm{DNA}} \rightleftharpoons {\mathrm{IG}}4{\mathrm{\ }} \rightleftharpoons {\mathrm{HG}}4$), but the difference in fitting was minimal (Supplementary Fig. S5C). Given that HG4 R-loops exhibit greater stability than IG4 R-loops [61, 62], and the reverse conversion from HG4 to IG4 appears negligible, we excluded this step from our model. One limitation is that our model is based on a 30-min endpoint, which may miss slower or higher-order transitions. Although we performed transcription reactions up to 45 min, we excluded this time point from analysis due to plateaued transcription caused by rNTP depletion. However, we continued to observe IG4 R-loop to HG4 R-loop conversion at 45 min (Supplementary Fig. S9A). While this model is a simplification and may not fully capture all aspects of the process, it allows us to analyze the relationship between IG4 and HG4 formation rates and assess how different PQS sequences influence these dynamics.

In our experiments with the 11 construct, which cannot form an IG4 R-loop, we observed a faint band migrating at the same position as the IG4 R-loop band. We hypothesize that, due to the G-rich nature of the sequence, it may form an incomplete G4 structure or another secondary structure. Additionally, we consistently observed a third band above the HG4 R-loop structure, emerging at later time points, typically after HG4 R-loop formation. Since this band had low intensity, we did not include it in our quantification. At this stage, we are unable to determine its exact nature, but it may represent a complex secondary structure involving an R-loop, G4, and RNA.

HG4 R-loops form later during transcription and exhibit distinct biophysical properties. Unlike IG4, HG4 R-loops are structurally more flexible and exhibit greater stability [61, 62]. HG4 can potentially form larger and more stable G4 structures through the participation of more than four G-tracts, contributed by both the DNA and RNA strands, thereby abrogating RNAP stalling. This enhanced flexibility and stability may facilitate RNA production by promoting the opening of the transcription bubble and alleviating DNA torsional constraints, thereby enabling more efficient RNAP progression.

Longer PQS loops favor HG4 R-loop formation and are associated with increased RNA production (Fig. 4). The reduced stability of longer loops may facilitate the conversion of IG4 R-loops to HG4 R-loops (Fig. 5). Additionally, the lower GC density of longer loops weakens R-loop stability, making them easier for RNAP to unwind and enabling successive transcription cycles. These contrasting effects highlight the critical role of PQS loop length in transcriptional regulation, influenced by the interplay between G4 stability and GC density, which affects R-loop stability. While our study focused on PQS constructs containing thymine (T) linkers for consistency, variation in linker composition—such as substituting adenine (A) for thymine—can alter G4 topology and R-loop formation patterns (Supplementary Fig. S9B and C).

In contrast, IG4 R-loops act as physical barriers (Supplementary Fig. S8), impeding RNAP progression and increasing torsional strain when formed on the non-template strand [63]. This strain arises because all G-tracts originate from the same DNA strand, which must twist and fold to form the G4 structure. The stability and inhibitory nature of IG4 R-loops are particularly pronounced in PQS sequences with short loop lengths. For example, among the PQS tested, the 111 PQS—with the highest GC content and the most stable IG4 R-loop structure—exhibited the lowest RNA production. The high GC density of short-loop PQS results in a stable R-loop that is difficult for RNAP to unwind, limiting transcription efficiency [64, 65]. However, in addition to loop length and GC density, the position of the PQS with respect to the promoter may also influence its regulatory effects, potentially affecting RNAP stalling, backtracking, or the efficiency of R-loop resolution. Further studies are needed to explore how PQS positioning modulates transcriptional outcomes.

Our findings suggest that HG4 R-loops play a significant role in transcriptional regulation. Given that HG4 R-loop formation is transcription-dependent, we hypothesize that HG4 R-loops may function as a positive feedback mechanism to enhance gene expression or as a sensor to modulate RNA levels in cells. The susceptibility of HG4 R-loops to RNase H digestion, which targets DNA:RNA hybrids (Supplementary Fig. S7C), along with the recent discovery of the transcription-coupled helicase CSB (Cockayne syndrome B protein), which preferentially unfolds intermolecular G4s over intramolecular ones [66], suggest that HG4 R-loops may be dynamically regulated or resolved by specific cellular factors.

While previous studies suggest a 1:3 RNA:DNA ratio may be optimal for HG4 formation, the structural variability and functional roles of HG4 require further investigation. These include understanding how HG4 interacts with proteins, responds to cellular ionic conditions, and contributes to transcriptional regulation within the cellular environment. Investigating the structural and functional diversity of HG4 will provide deeper insights into its role in genome regulation and broader cellular processes.

Our study highlights the contrasting roles of IG4 R-loops and HG4 R-loops in transcriptional regulation, emphasizing the importance of PQS features, such as loop length, in shaping their formation and effects. By elucidating the mechanisms underlying these structures, we provide new insights into how R-loop-associated G4s influence transcription. While our *in vitro* T7 RNAP transcription system provides valuable mechanistic insights, we acknowledge that this system lacks the chromatin context and regulatory elements present in cells and may not fully reflect endogenous G4-mediated processes. These findings enhance understanding of how PQS commonly found in the human genome may behave based on their sequence characteristics. Future research exploring the function of these structures in cellular contexts and their regulatory mechanisms could offer insights for developing therapeutic interventions targeting G4s.

## Supplementary Material

gkaf930_Supplemental_Files

## Data Availability

Custom codes are available on GitHub (https://github.com/Ha-SingleMoleculeLab) and Zenodo at the DOI: 10.5281/zenodo.16990017 and https://doi.org/10.5281/zenodo.16990017.
