# Supplementary material for: Opposing roles of two R-loop associated G-quadruplexes in tuning transcription activity
Source: Nucleic Acids Res. 2025 Sep 23;53(18):gkaf930. doi: 10.1093/nar/gkaf930 (PMC12455608; doi:10.1093/nar/gkaf930)
Supplement: gkaf930_Supplemental_Files [file gkaf930_supplemental_files.zip › SUPPLEMENTARY INFORMATION.docx]

**Table 1.** All sequences used

| **Construct name** | **top sequence (5'-3')** | **bottom sequence (5'-3')** |
| --- | --- | --- |
| **1** | G GGA GAC CAC AAC GTT A GGG T GGG T ATC AGC TCC AGG T | ACC TGG AGC TGA TA CCC A CCC TAA CGT TGT GGT CTC CC |
| **11** | G GGA GAC CAC AAC GTT A GGG T GGG T GGG T ATC AGC TCC AGG T | ACC TGG AGC TGA TA CCC A CCC A CCC TAA CGT TGT GGT CTC CC |
| **111** | G GGA GAC CAC AAC GTT A GGG T GGG T GGG T GGG T ATC AGC TCC AGG T | ACC TGG AGC TGA TA CCC A CCC A CCC A CCC TAA CGT TGT GGT CTC CC |
| **112** | G GGA GAC CAC AAC GTT A GGG T GGG T GGG TT GGG T ATC AGC TCC AGG T | ACC TGG AGC TGA TA CCC AA CCC A CCC A CCC TAA CGT TGT GGT CTC CC |
| **121** | G GGA GAC CAC AAC GTT A GGG T GGG TT GGG T GGGT ATC AGC TCC AGG T | ACC TGG AGC TGA TA CCC A CCC AA CCC A CCC TAA CGT TGT GGT CTC CC |
| **122** | G GGA GAC CAC AAC GTT A GGG T GGG TT GGG TT GGG T ATC AGC TCC AGG T | ACC TGG AGC TGA TA CCC AA CCC AA CCC A CCC TAA CGT TGT GGT CTC CC |
| **123** | G GGA GAC CAC AAC GTT A GGG T GGG TT GGG TTT GGG T ATC AGC TCC AGG T | ACC TGG AGC TGA TA CCC AAA CCC AA CCC A CCC TAA CGT TGT GGT CTC CC |
| **133** | G GGA GAC CAC AAC GTT A GGG T GGG TTT GGG TTT GGG T ATC AGC TCC AGG T | ACC TGG AGC TGA TA CCC AAA CCC AAA CCC A CCC TAA CGT TGT GGT CTC CC |
| **144** | G GGA GAC CAC AAC GTT A GGG T GGG TTTT GGG TTTT GGG T ATC AGC TCC AGG T | ACC TGG AGC TGA TA CCC AAAA CCC AAAA CCC A CCC TAA CGT TGT GGT CTC CC |
| **211** | G GGA GAC CAC AAC GTT A GGG TT GGG T GGG T GGG T ATC AGC TCC AGG T | ACC TGG AGC TGA TA CCC A CCC A CCC AA CCC TAA CGT TGT GGT CTC CC |
| **212** | G GGA GAC CAC AAC GTT A GGG TT GGG T GGG TT GGG T ATC AGC TCC AGG T | ACC TGG AGC TGA TA CCC AA CCC A CCC AA CCC TAA CGT TGT GGT CTC CC |
| **221** | G GGA GAC CAC AAC GTT A GGG TT GGG TT GGG T GGG T ATC AGC TCC AGG T | ACC TGG AGC TGA TA CCC A CCC AA CCC AA CCC TAA CGT TGT GGT CTC CC |
| **222** | G GGA GAC CAC AAC GTT A GGG TT GGG TT GGG TT GGG T ATC AGC TCC AGG T | ACC TGG AGC TGA TA CCC AA CCC AA CCC AA CCC TAA CGT TGT GGT CTC CC |
| **231** | G GGA GAC CAC AAC GTT A GGG TT GGG TTT GGG T GGG T ATC AGC TCC AGG T | ACC TGG AGC TGA TA CCC A CCC AAA CCC AA CCC TAA CGT TGT GGT CTC CC |
| **233** | G GGA GAC CAC AAC GTT A GGG TT GGG TTT GGG TTT GGG T ATC AGC TCC AGG T | ACC TGG AGC TGA TA CCC AAA CCC AAA CCC AA CCC TAA CGT TGT GGT CTC CC |
| **312** | G GGA GAC CAC AAC GTT A GGG TTT GGG T GGG TT GGG T ATC AGC TCC AGG T | ACC TGG AGC TGA TA CCC AA CCC A CCC AAA CCC TAA CGT TGT GGT CTC CC |
| **1111** | G GGA GAC CAC AAC GTT A GGG T GGG T GGG T GGG T GGG T ATC AGC TCC AGG T | ACC TGG AGC TGA TA CCC A CCC A CCC A CCC A CCC TAA CGT TGT GGT CTC CC |
| **11111** | G GGA GAC CAC AAC GTT A GGG T GGG T GGG T GGG T GGG T GGG T ATC AGC TCC AGG T | ACC TGG AGC TGA TA CCC A CCC A CCC A CCC A CCC A CCC TAA CGT TGT GGT CTC CC |
| **333** | G GGA GAC CAC AAC GTT A GGG TTT GGG TTT GGG TTT GGG T ATC AGC TCC AGG T | ACC TGG AGC TGA TA CCC AAA CCC AAA CCC AAA CCC TAA CGT TGT GGT CTC CC |
| **A111** | G GGA GAC CAC AAC GTT A GGG A GGG A GGG A GGG T ATC AGC TCC AGG T | ACC TGG AGC TGA TA CCC T CCC T CCC T CCC TAA CGT TGT GGT CTC CC |
| **A222** | G GGA GAC CAC AAC GTT A GGG AA GGG AA GGG AA GGG T ATC AGC TCC AGG T | ACC TGG AGC TGA TA CCC TT CCC TT CCC TT CCC TAA CGT TGT GGT CTC CC |


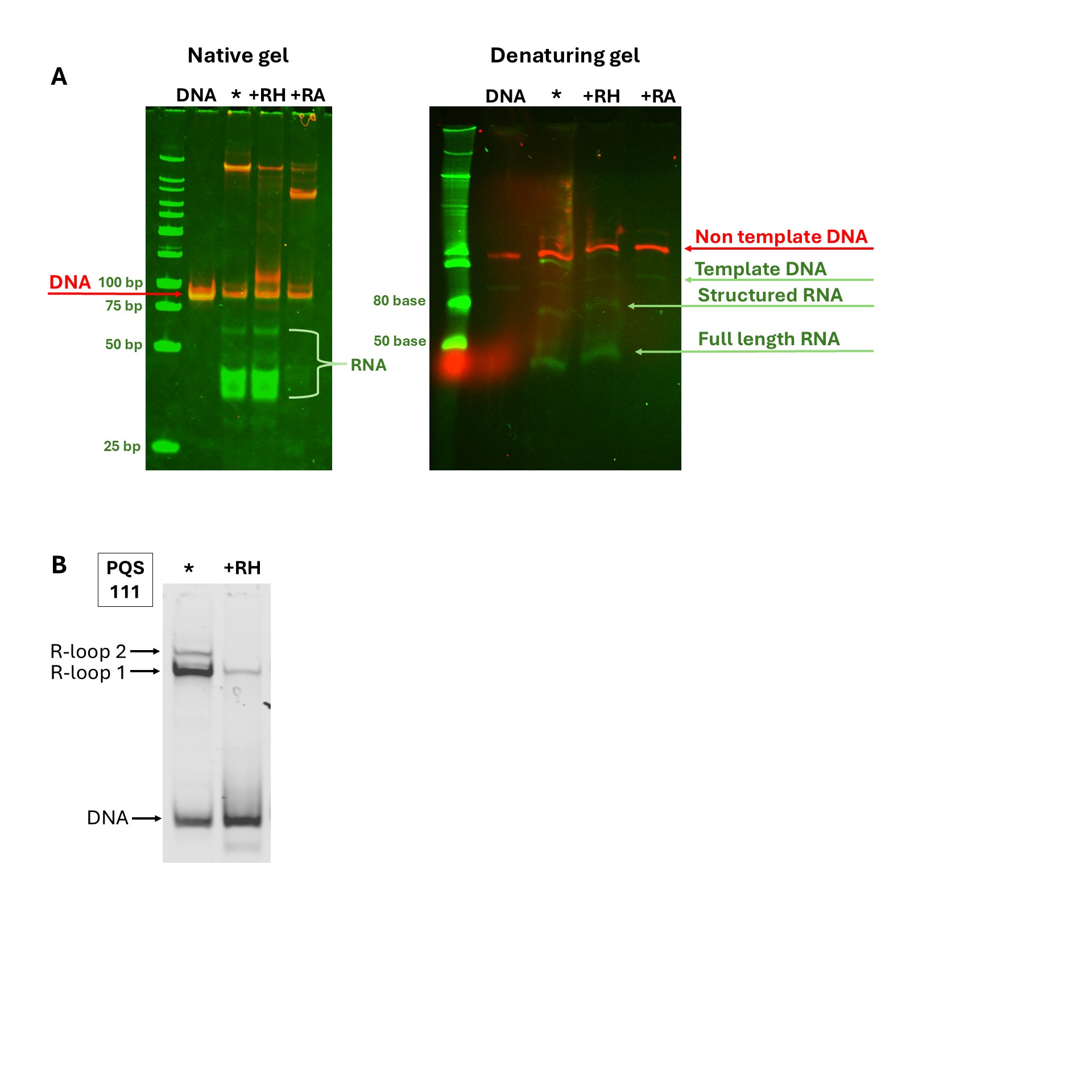


**Supplementary Fig. 1. Smeared RNA band corresponds to RNA folded into secondary structures and verification of R-loop identity** (A) Left: Native PAGE EMSA. Lane 1: PQS 111 DNA only. Lane 2: Transcription of PQS 111 for 20 minutes. Lane 3: RNaseH digestion post-transcription. Lane 4: RNase A digestion post-transcription. DNA bands are shown in red; RNA bands are stained green. Right: Denaturing gel of the same samples to resolve smeared RNA. The red band corresponds to the non-template DNA; green bands represent either template DNA or RNA. (B) Left: Transcription of PQS construct under standard conditions for 30 minutes. Right: RNase H digestion for 5 minutes post-transcription. Asterisk (*) denotes standard transcription conditions.

**
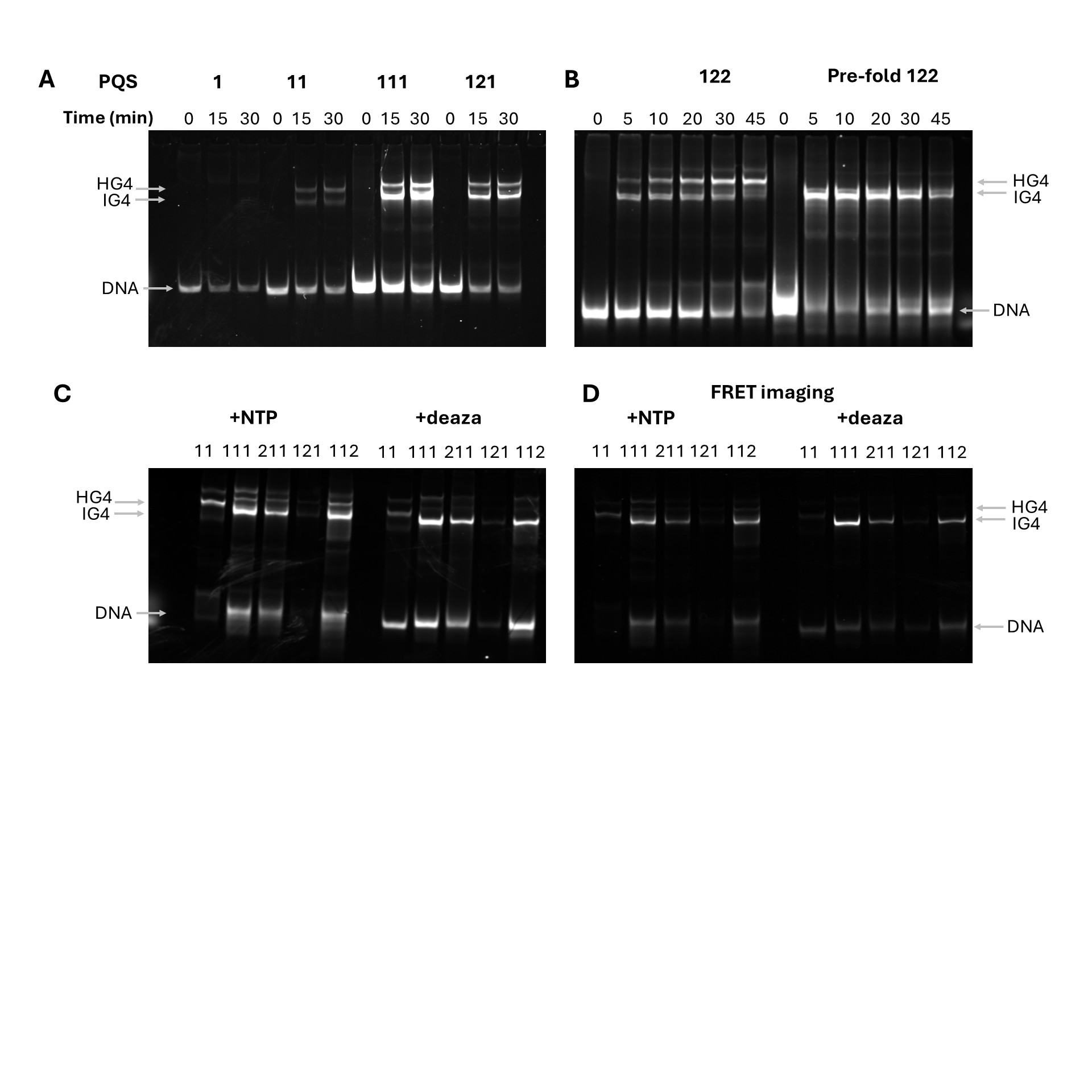
**

**Supplementary Fig. 2. Additional experiments validating the identities of R-loop 1 as an IG4 R-loop and R-loop 2 as an HG4 R-loop.** (A) EMSA showing transcription time course (0, 15, 30 min) for 1, 11, 111, and 121 constructs. (B) EMSA comparing R-loop formation using standard duplex PQS 121 DNA versus pre-folded 121 G4 construct. (C) EMSA of transcription with standard rNTPs versus deaza-rGTP substitution, which inhibits G4 formation, for various PQS constructs. (D) FRET-based EMSA imaging of G4 formation across different PQS sequences (Cy3 excitation, Cy5 emission, 1 min exposure).


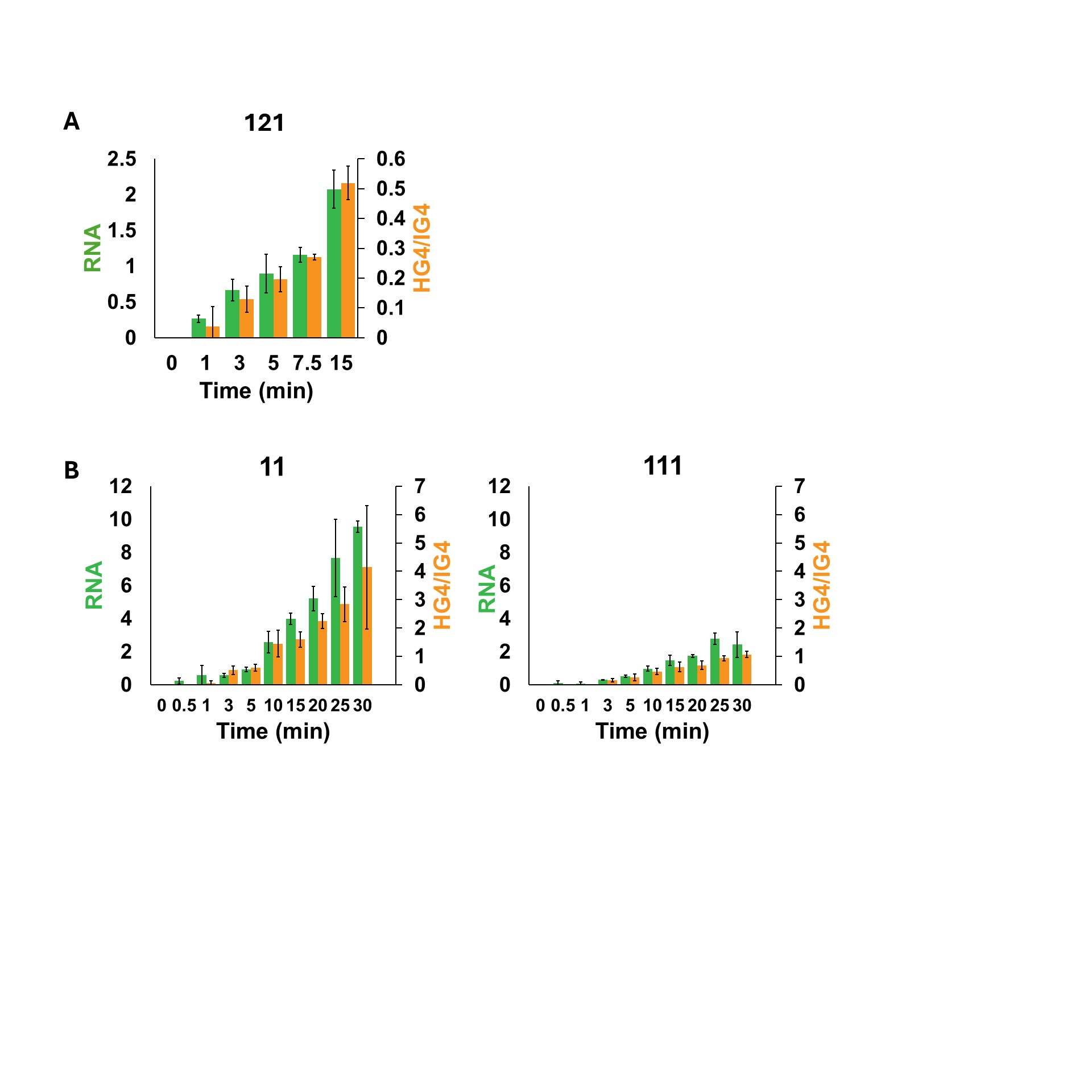


**Supplementary Fig 3. RNA levels correlate with the HG4/IG4** (A) Two axis plot showing correlation between RNA levels and the HG4/IG4 ratio for PQS 121. (B) Two axis plot showing correlation between RNA levels and HG4/IG4 ratio for PQS 111 and 11 constructs. All data represent the mean of three independent replicates with standard deviations shown. Raw values are available in the data source file.


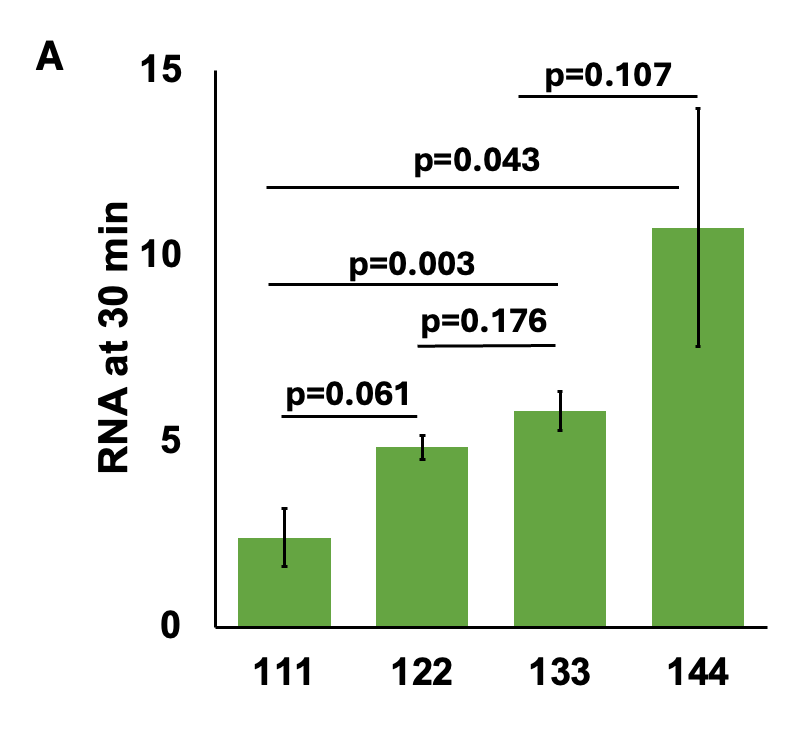


**Supplementary Fig 4. Longer loop lengths correlate with increased RNA production at 30 minutes.** (A) Quantification of RNA levels at 30 minutes for constructs 111, 122, 133, and 144. p-values from two-tailed t-test are indicated. Data represent the mean ± standard deviation from three independent replicates. Raw values are available in the data source file.


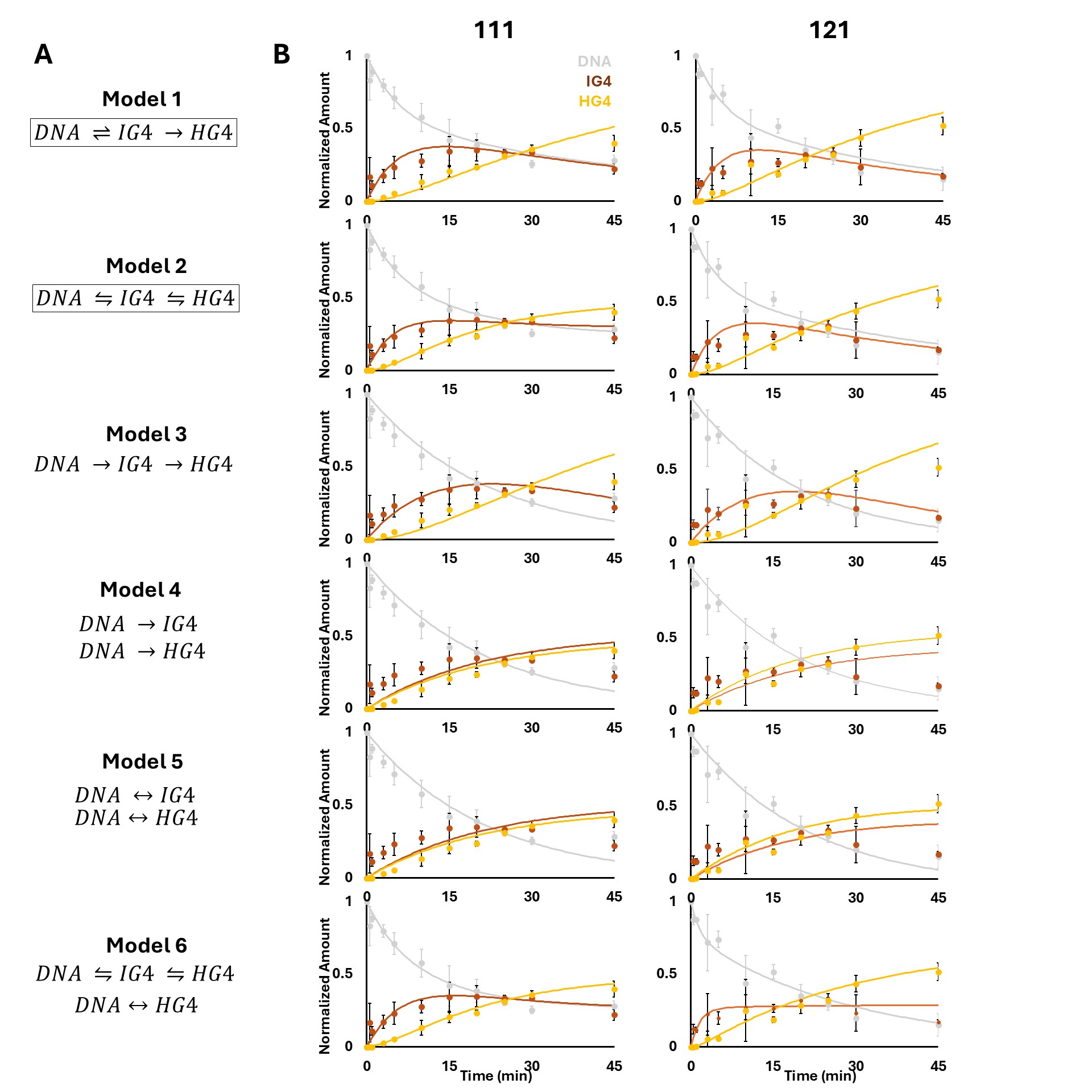


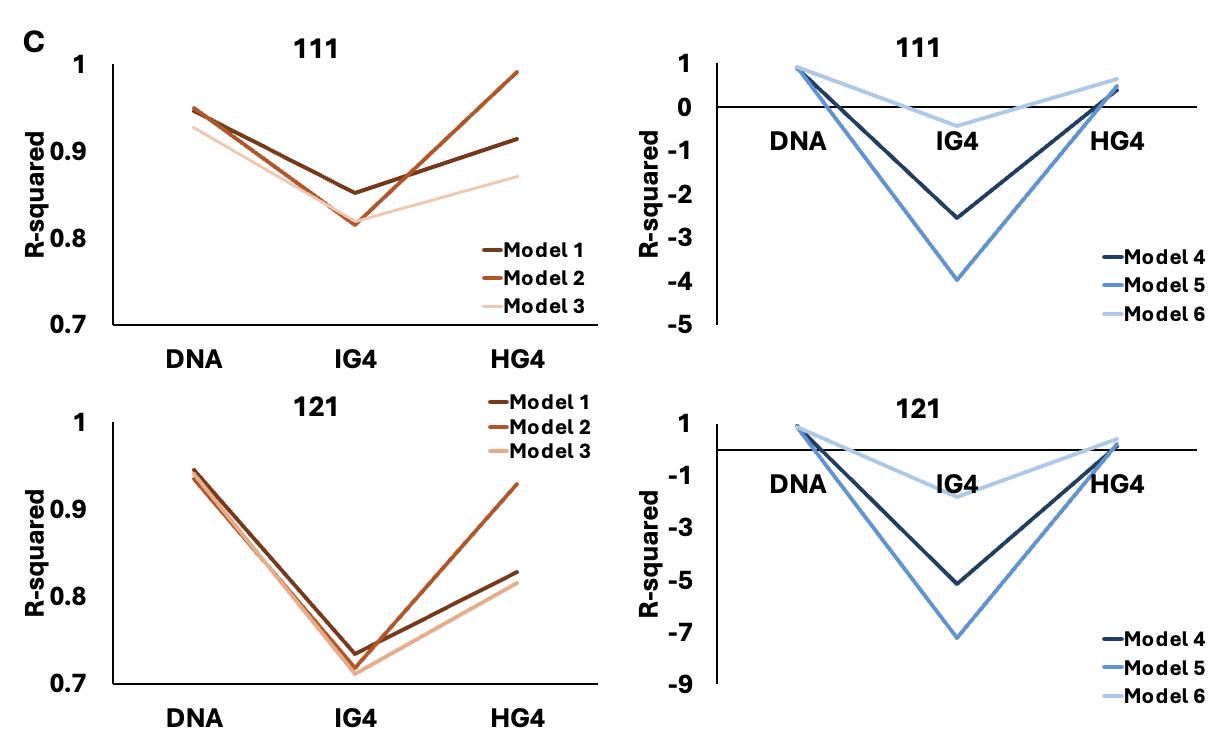


**Supplementary Fig 5. Model 1 provides the best fit to experimental data** (A) List of kinetic models tested for fitting. (B) Representative fitting results for PQS 111 and 121 constructs. Models 1 and 2 best capture the experimental trends. (C) Comparison of R² values across models. While Models 1–3 fit the data comparably well, Models 4-6 show poor fit, as indicated by negative R² values.


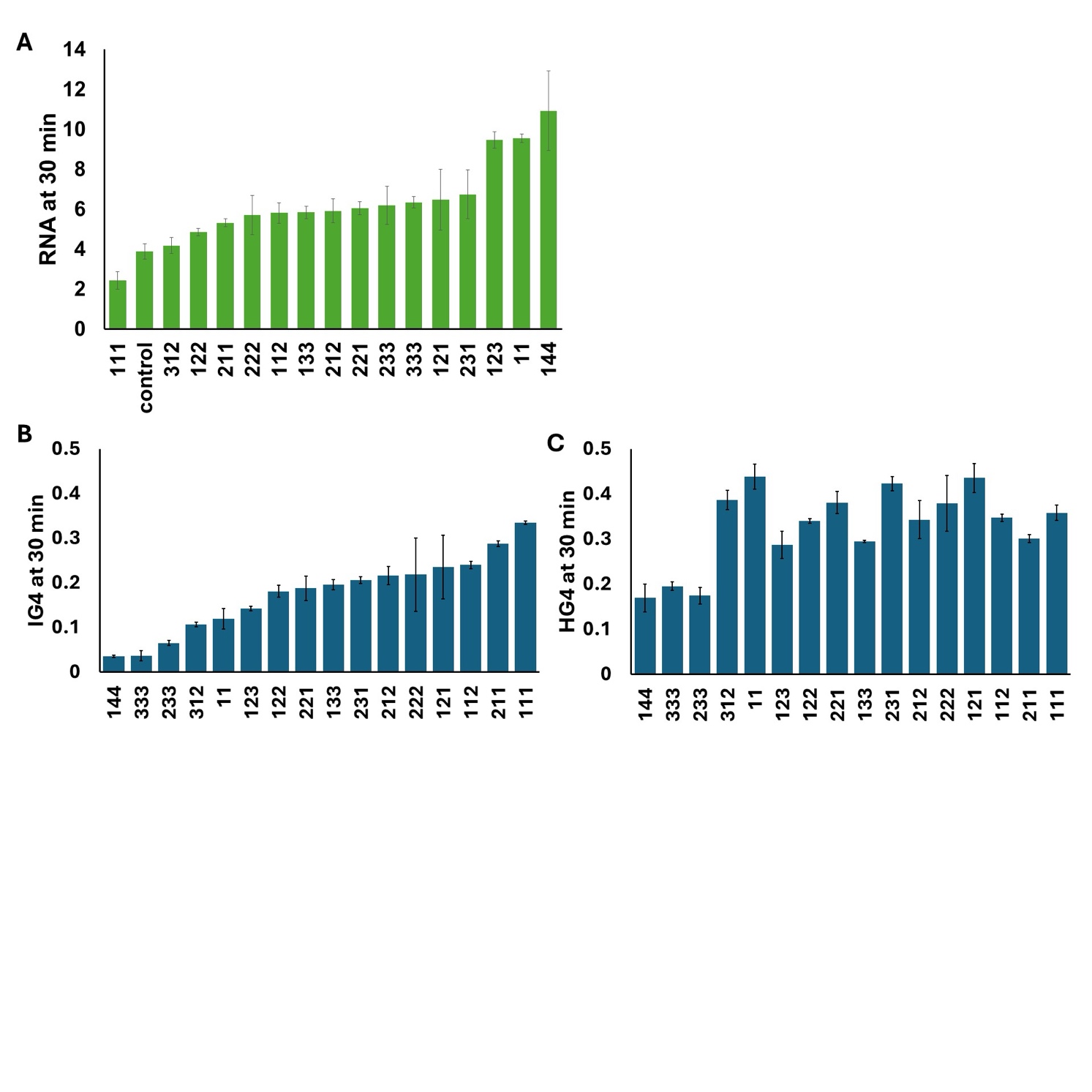


**Supplementary Fig. 6. PQS sequence influences RNA output and G4 dynamics.**

(A) Ranking of RNA production at the 30-minute time point across all tested constructs. (B) Ranking of IG4 R-loop levels at 30 minutes. (C) Ranking of HG4 R-loop levels at 30 minutes. In all panels, bars represent the mean of three independent replicates; error bars indicate the standard error of the mean (SEM). Raw values are available in the data source file.

**
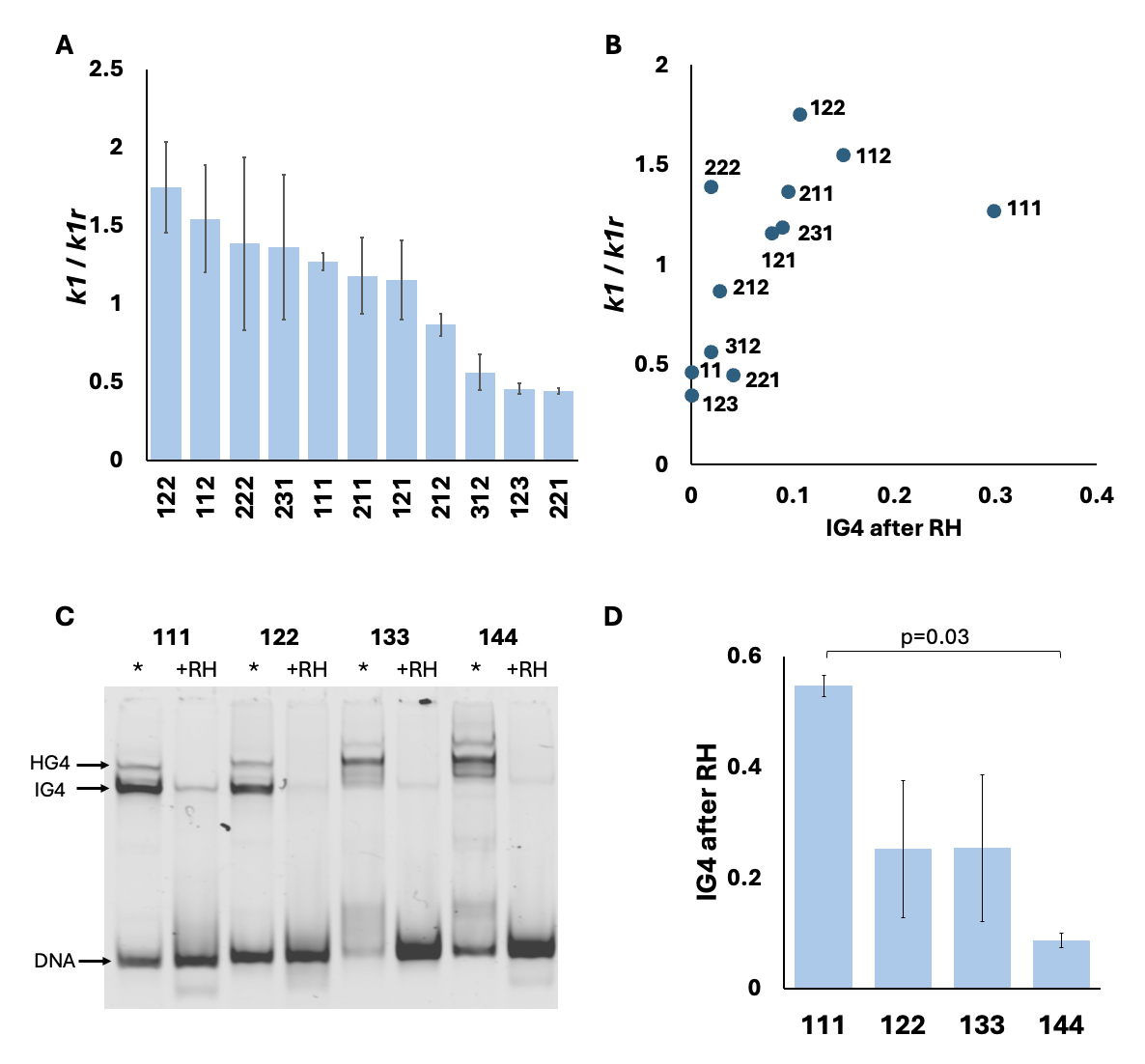
**

**Supplementary Fig. 7. RNase H resistance as a proxy for IG4 stability**

(A) Ranking of k_1/k_{1r} values across different PQS sequences, derived from kinetic modeling using the chem_kinetics Python package. (B) Positive linear correlation between normalized IG4 band intensity after RNase H digestion and the corresponding k_1/k_{1r} values. (C) EMSA of transcription products after 20 minutes under normal conditions (lane marked *). Lane marked +RH was treated with RNase H for 5 minutes post-transcription. Two independent replicates were performed. (D) Quantification of IG4 band intensity remaining after RNase H digestion, normalized to pre-digestion levels, for constructs 111, 122, 133, and 144. A statistically significant difference is observed between 111 and 144 (two-tailed t-test). For all bar graphs, bars represent the mean of three independent replicates; error bars indicate the standard error of the mean (SEM). Raw values are available in the data source file.


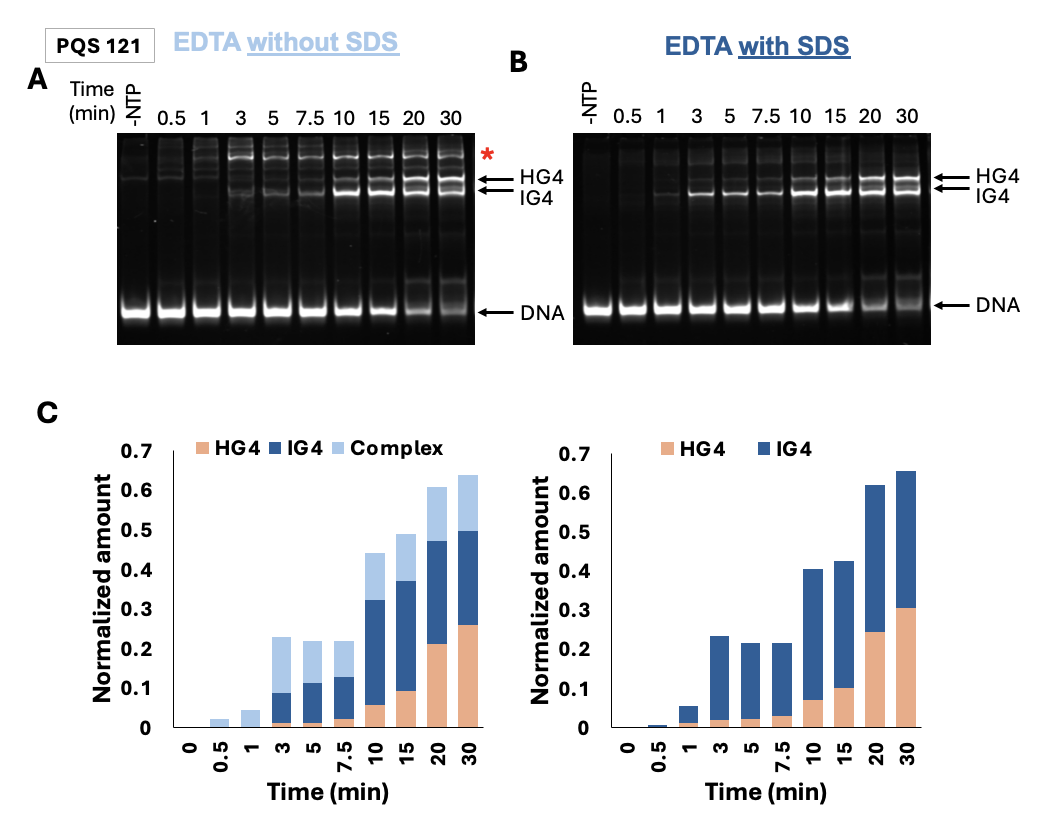


**Supplementary Fig 8.** **RNAP associated with the IG4 R-loop.** (A) EMSA of the PQS 121 following transcription termination with EDTA alone. An upshifted band (*) indicates an RNAP-DNA complex. (B) EMSA of PQS 121 sequence where transcription was terminated with both EDTA and SDS. The upshifted band disappears, suggesting the dissociation of RNAP from the DNA. (C) Quantification of band intensities shows that the sum of the IG4 and complex bands in the EDTA-only condition equals the IG4 band in the EDTA + SDS condition. HG4 intensity remains unchanged between conditions. Raw values are available in the data source file.


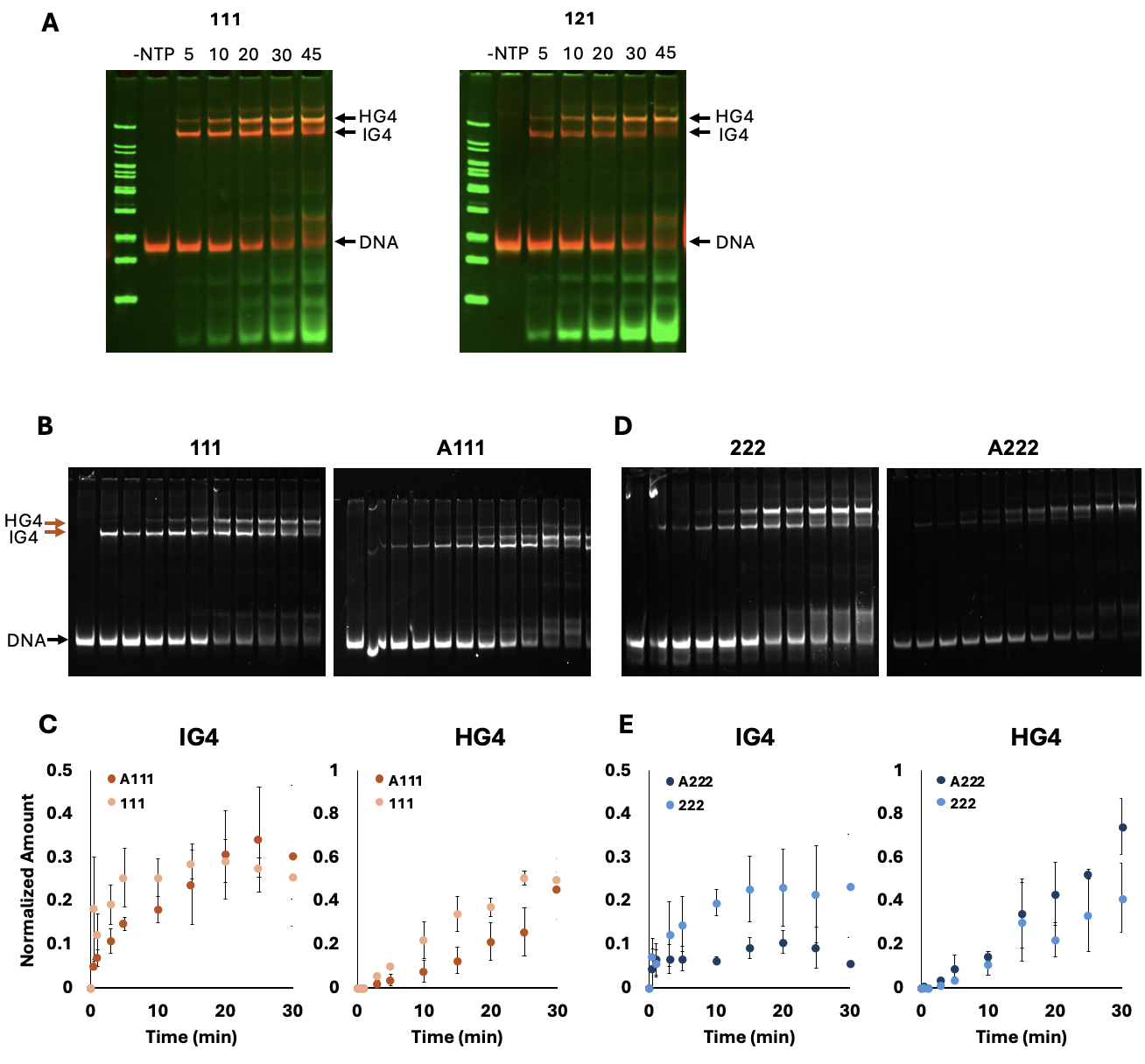


**Supplementary Fig. 9. R-loop formation dynamics at extended transcription time and linker identity comparison.** (A) EMSA of transcription with PQS constructs extended to 45 minutes. DNA is shown in red; RNA is shown in green. (B) EMSA comparing R-loop formation between PQS constructs with thymine (T) linkers (left) and adenine (A) linkers (right) for PQS 111 and A111. Red arrows indicate R-loop bands; black arrows indicate DNA. (C) Quantification of IG4 and HG4 band intensities for PQS 111 and A111. Values represent the mean of three independent replicates; error bars indicate standard deviation. (D) EMSA comparing R-loop formation between PQS constructs with thymine (T) linkers (left) and adenine (A) linkers (right) for PQS 222 and A222. Red arrows indicate R-loop bands; black arrows indicate DNA. (E) Quantification of IG4 and HG4 band intensities for PQS 222 and A222. Values represent the mean of three independent replicates; error bars indicate standard deviation. Raw data are available in the data source file.
